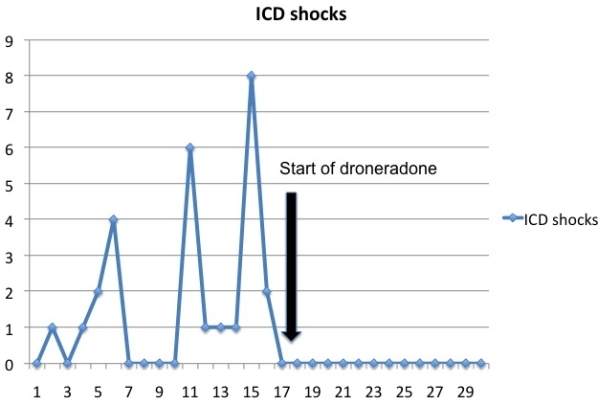# Dronedarone for Recurrent Ventricular Tachycardia: A Real Alternative?

**DOI:** 10.1016/s0972-6292(16)30483-1

**Published:** 2012-04-30

**Authors:** Victor Exposito, Felipe Rodriguez-Entem, Susana Gonzalez-Enriquez, Juan Jose Olalla

**Affiliations:** Arrhytmia Unit. Cardiology Service. Universitary Hospital Marques de Valdecilla. Santander. Spain.

**Keywords:** Ventricular Tachycardia, Dronedarone

## Abstract

Sustained ventricular tachycardia (VT) is an important cause of morbidity and sudden death in patients with dilated cardiomyopathy. Although ICD effectively terminate VT episodes and improve survival, shocks reduce quality of life, and episodes of VT predict increased risk of heart failure and death despite effective therapy. Patients suffering recurrent VT episodes remain a challenge. Antiarrhytmic therapy reduces VT episodes, but it is associated with serious adverse events, and disappointing efficacy. Catheter ablation has emerged as an important option to control recurrent VT, but major procedure-related complications, and even death, are still issues to concern. And even with these armamentaria, some patients still have recurrent VT episodes and ICD shocks. We report on a patient with non-ischemic dilated cardiomyopathy and recurrent ventricular tachycardia resistant to multiple antiarrhytmic agents, in whom dronedarone was effective in completely suppressing ventricular tachycardia episodes.

## Background

Sustained ventricular tachycardia (VT) is an important cause of morbidity and sudden death in patients with dilated cardiomyopathy. Although ICDs effectively terminate VT episodes and improve survival, shocks reduce quality of life, and episodes of VT predict increased risk of heart failure and death despite effective therapy. Patients suffering recurrent VT episodes remain a challenge. Antiarrhythmic therapy reduces VT episodes, but it is associated with serious adverse events, and disappointing efficacy. Catheter ablation has emerged as an important option to control recurrent VT, but major procedure-related complications, and even death, are still issues of concern. And even with this armamentarium, some patients still have recurrent VT episodes and ICD shocks. We report on a patient with non-ischemic dilated cardiomyopathy and recurrent ventricular tachycardia resistant to multiple antiarrhythmic agents, in whom dronedarone was effective in completely suppressing ventricular tachycardia episodes.

## Case Report

An 81-year-old man with non-ischemic dilated cardiomyopathy and severe ventricular systolic dysfunction (EF 30%) underwent biventricular ICD implantation (St Jude Promote AccelTM), after suffering sudden cardiac death due to ventricular tachycardia. Baseline rhythm was atrial fibrillation with slow ventricular response. He was on amiodarone, beta-blocker and angiotensin receptor blocker at the time of implantation, without heart failure symptoms.

The patient continued to have symptomatic VT episodes with appropriate ICD discharges. Amiodarone was increased, but it was discontinued due to severe hypothyroidism. He was placed on sotalol 80 mg twice a day, but soon stopped after patient developed QT prolongation and asthenia. Patient was switched to propafenone 300 mg 3 times daily, but again side effects (diarrhea, nausea-vomiting) led to therapy discontinuation. Symptomatic VT episodes with multiple morphologies requiring ICD discharges and/or emergency room visits continued, despite optimization of ATP therapy.

Procainamide was initiated with better tolerance, although patient continued to be symptomatic with frequent VT episodes. At this point in time, an echocardiogram showed marked left ventricular systolic function impairment (EF 15%). Of note, cardiac function in consecutive echocardiograms had initially remained stable after ICD implantation. The rest of pharmacological agents had not been modified. After myocardial ischemia was ruled out, a catheter ablation procedure was planned, but patient refused invasive procedures. Trying dronedarone was offered, with clear understanding of its off-label and compassionate use. After informed consent, patient was switched to droneradone 400 mg twice daily. On follow-up, patient reported significant improvement of his symptoms, with just one episode of VT successfully treated with ATP therapy over the next 12 months, and systolic function recovery to prior 30% coinciding with the decrease of arrhythmic burden. Significant reduction of ICD shocks before and after droneradone initiation is shown in Figure 1.

## Discussion

Droneradone represents the latest generation of antiarrhythmic drugs, a multichannel blocking agent with molecular structure similar to amiodarone, but with a more favorable safety profile, as the drug is no iodinated and has less lipophilicity [1], The possibility to overcome extracardiac effects of long-term treatment with amiodarone in AF patients seemed particularly promising, as clinical studies had shown that dronedarone effectively reduces ventricular rate and may prevent or delay the recurrence of AF. Even more, the ATHENA trial showed significant reductions in a composite end point of all-cause mortality and cardiovascular hospitalization with dronedarone use, and a post hoc analysis of the ATHENA data also suggested a decrease in stroke risk with this agent [2,3]. This initial enthusiastic state drove to droneradone incorporation into American College of Cardiology Foundation (ACCF)/American Heart Association (AHA)/Heart Rhythm Society (HRS), and European Society of Cardiology (ESC) 2010 guidelines on AF, even as a drug of first-line treatment in the latter [4]. Unfortunately, droneradone is clearly less effective in maintaining sinus rhythm than amiodarone, and, although significantly safer, recently FDA and EMA recommended warnings about possible risk of severe liver injury.

Dronedarone has not been specifically studied for patients with ventricular arrhythmias. However, animal studies have demonstrated antiarrhytmic properties on ventricular myocardium, and clinical experience may suggest it may be an alternative in selected cases [5,6]. In ATHENA trial patients on droneradone showed a reduction in arrhythmic death. Kowey et al reported a trend towards reduction in appropriate ICD shocks in patients on droneradone at high doses. Although this indication is not established, positive experience is accumulating in this setting. Fink et al reported suppression of VT episodes as read in device memory over 6 months follow-up in a patient with paroxysmal AF, ischemic cardiomyopathy and ICD implantation due to ventricular arrhythmias. Dronedarone was started as alternative to amiodarone because of intolerance [7]. Finally, Shaaraoui et al described recently suppression of recurrent ventricular tachycardia refractory to multiple drug therapy and catheter ablation in a DCM patient with mild ventricular dysfunction and no signs of heart failure [8].

Use of droneradone in severely depressed ventricular function remains controversial, as its use in congestive heart failure patients may lead to worsening symptoms and even cardiogenic shock [9]. In the ANDROMEDA trial, dronedarone was associated with increased mortality when tested in New York Heart Association (NYHA) III/IV patients with left ventricular ejection fractions of less than 35%, who also had a recent hospitalization for decompensated heart failure.

In our case, droneradone suppressed dramatically the number of VT episodes, without impairing heart failure symptoms, and even restoring ventricular function to baseline, presumably due to the mid-term adverse consequences of frequent ventricular ectopy and runs of non-sustained and sustained VT.

Although no definitive conclusions can be made with so few cases, the notion of a potential role for droneradone in controlling ventricular arrhythmias merits further exploration.

## Figures and Tables

**Figure 1:**